# Transjugular Leadless Pacemaker Implantation in an Adolescent With Central Hypoventilation Syndrome: A Case Report

**DOI:** 10.1111/pace.70146

**Published:** 2026-01-26

**Authors:** Marzia Giaccardi, Gulio Porcedda, Zaccaria Ricci, Hendrik Bonnemeier, Benito Baldauf

**Affiliations:** ^1^ Cardiology Department Santa Maria Annunziata Hospital Florence Italy; ^2^ Meyer Children's Hospital, IRCCS Florence Italy; ^3^ Christian‐Albrechts University Kiel Kiel Germany; ^4^ University of Applied Sciences Bremerhaven Germany; ^5^ Asklepios Hospital Schildautal Seesen Seesen Germany

**Keywords:** case report, congenital central hypoventilation syndrome, internal jugular vein, leadless pacemaker, pediatric pacing

## Abstract

**Background:**

Permanent pacing in pediatric patients is complicated by small body size, vascular access limitations, and the need for durable long‐term management. Leadless pacemakers offer an emerging alternative that minimizes infection and lead‐related complications, yet vascular access remains a key challenge in young patients.

**Case Presentation:**

We report a 14‐year‐old male with congenital central hypoventilation syndrome (CCHS; PHOX2B polyalanine repeat mutation 20/26) who presented with recurrent nocturnal oxygen desaturation secondary to prolonged sinus pauses despite tracheostomy‐assisted mechanical ventilation. Continuous implantable loop recorder monitoring confirmed pauses of up to 10 s, temporally associated with desaturation events. Given the small caliber of the femoral veins and the need for long‐term pacing, a leadless pacemaker was implanted via the right internal jugular vein. Device positioning was optimized under fluoroscopic guidance to achieve stable septal fixation with excellent electrical parameters (sensing: 9 mV; threshold 0.5 V at 0.24 ms; impedance: 530 Ω). The procedure and recovery were uneventful.

**Follow‐up:**

At 1‐year follow‐up, pacing burden was 1% with stable sensing and threshold parameters, and no recurrent desaturation episodes were observed.

**Conclusion:**

This case highlights the complex interplay between arrhythmia and oxygen desaturation in CCHS, illustrating how permanent pacing can restore both rhythm and ventilatory stability. Transjugular leadless pacemaker implantation represents a safe and effective option for adolescents when femoral access is limited. In younger patients, careful anatomical assessment, awareness of long‐term device management, and individualized selection between retrievable and established systems are essential to optimize outcomes.

## 1 Introduction

Pacemaker implantation in pediatric patients presents unique challenges due to limited venous access, ongoing somatic growth, and the need for devices that ensure long‐term durability with minimal complications. Traditional epicardial and transvenous pacing systems remain standard but carry well‐known risks such as lead fracture, infection, venous obstruction, and the need for repeated surgical revisions due to battery depletion.

Leadless pacemakers overcome many of these limitations by eliminating leads and generator pockets, thereby reducing infection risk and mechanical complications. Their use in children, however, is still uncommon, primarily because of the large sheath sizes required for implantation. When femoral access is limited by vessel caliber, the internal jugular approach provides a viable alternative route, as described in several small pediatric series [1, 2, 3, 4].

In addition, the use of leadless pacemakers in children raises unresolved questions regarding lifetime device management, including the potential need for implantation of multiple devices and the feasibility of device retrieval after very long dwelling times. Even for retrievable systems, long‐term extraction data in pediatric populations remain limited.

Congenital central hypoventilation syndrome (CCHS) is a rare genetic disorder of autonomic nervous system regulation, most commonly caused by mutations in the PHOX2B gene [5]. It is characterized by impaired central respiratory drive, particularly during sleep, and may be accompanied by cardiovascular autonomic dysfunction, including sinus node dysfunction and bradyarrhythmias. These cardiac manifestations may contribute to hypoxemia independently of central apnea, yet remain underrecognized in clinical practice.

We report the case of an adolescent with CCHS who exhibited sinus pauses leading to significant oxygen desaturation during sleep. Following detailed assessment of venous anatomy and pacing options, the patient underwent successful leadless pacemaker implantation via the internal jugular vein

## 2 Case Presentation

A 14‐year‐old male with CCHS (PHOX2B polyalanine repeat mutation 20/26) and tracheostomy‐dependent nocturnal ventilation was referred for evaluation of recurrent nocturnal oxygen desaturation. Continuous implantable loop recorder monitoring demonstrated sinus pauses up to 10 s, temporally associated with the onset of desaturation episodes. These findings suggested that bradyarrhythmia, rather than central apnea alone, contributed to the patient's nocturnal hypoxemia.

Past medical history: Precocious puberty (treated with GnRH analog until 2020), hyperinsulinism (on non‐diuretic benzothiadiazine), and hypertension (amlodipine 7.5 mg/day). Echocardiography revealed normal left ventricular function (LVEF 54%) without structural heart disease. Body height: 173 cm; Body weight: 73 kg; BMI: 24.39 (overweight >23.01 for adolescents); BSA: 1.8 m^2^.

**FIGURE 1:**
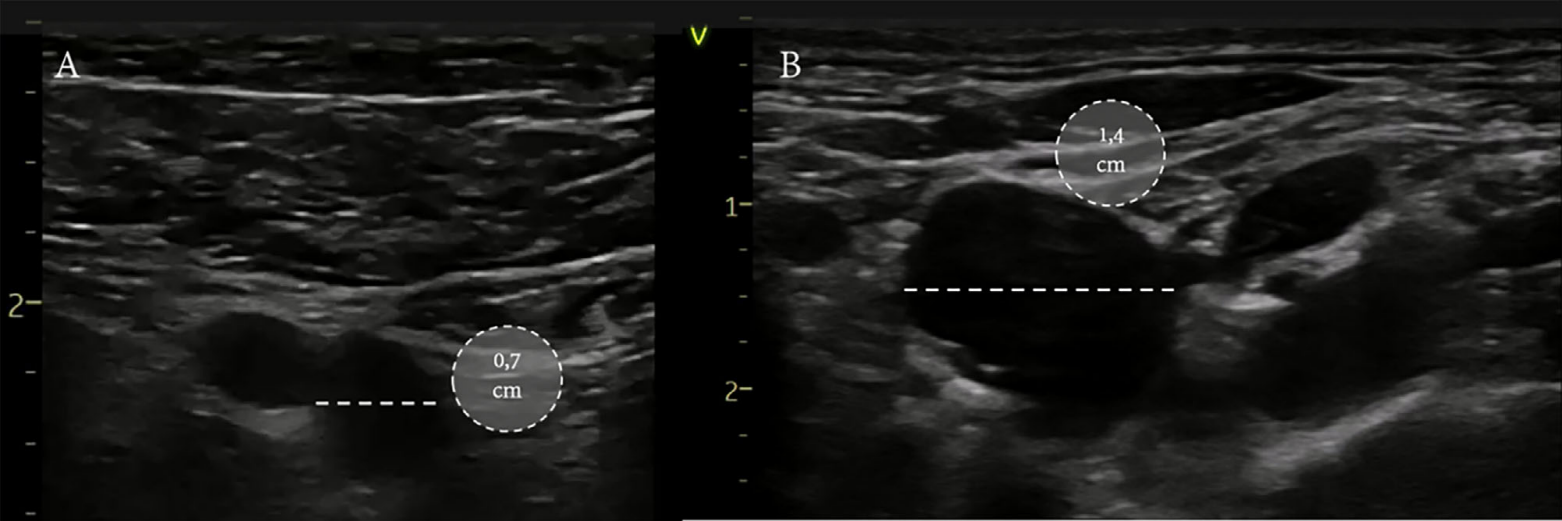
Ultrasound assessment of the right femoral vein (left: panel A) and right internal jugular vein (right: panel B) to evaluate vascular access for cardiac catheterization. [Colour figure can be viewed at wileyonlinelibrary.com]

Pre‐procedural assessment: Venous imaging showed bilaterally small femoral veins (∼7 mm), unsuitable for large‐caliber delivery systems (Figure 1). The right internal jugular vein was patent and of sufficient diameter for access (∼14 mm). After multidisciplinary discussion and informed consent, the team elected to proceed with leadless pacemaker implantation via the right internal jugular route.

## 3 Device Selection

Considering the patient's young age and long‐term pacing needs, a helix‐fixated (active‐fixation) leadless pacemaker was initially evaluated for its retrievability and dual‐chamber potential. However, at the time of intervention, it was not yet commercially available in our region. Given extensive operator experience and the robust clinical evidence supporting its safety and performance, a tine‐fixated (passive‐fixation) leadless pacemaker with extensive market experience was chosen, balancing procedural familiarity, immediate safety, and long‐term reliability despite its potentially limited retrievability.

## 4 Procedure

**FIGURE 2:**
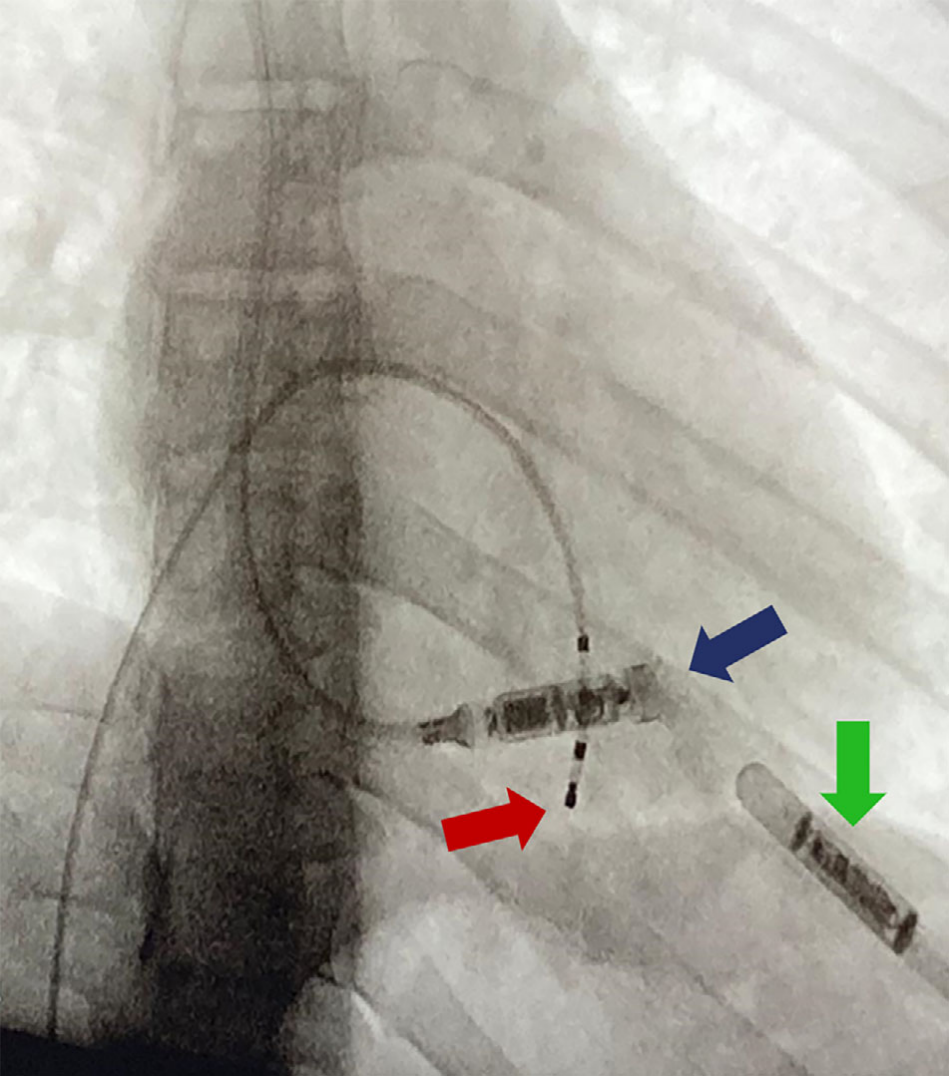
Radiographic image showing the leadless pacemaker delivery catheter and the pacemaker in situ (blue arrow), demonstrating accurate placement within the target cardiac structure. The red arrow indicates the quadripolar temporary pacing catheter, and the green arrow identifies the implantable loop recorder. [Colour figure can be viewed at wileyonlinelibrary.com]

Under general anesthesia, right internal jugular access was obtained using ultrasound guidance. During induction, the patient developed a prolonged sinus arrest requiring temporary pacing. A 23 French delivery catheter was advanced into the right ventricle under fluoroscopic guidance. A small amount of contrast confirmed septal orientation, and the device was implanted in the mid‐inferoapicoseptal region. While individual tines were not distinctly visualized angiographically, device stability was confirmed by tug testing and stable electrical parameters. Electrical testing demonstrated stable parameters (sensing 10 mV; threshold 0.5 V at 0.24 ms; impedance 600 Ω). Post‐deployment imaging confirmed optimal position and stable fixation (Figure 2).

## 5 Postoperative Course

The patient remains tracheostomy‐dependent for nocturnal ventilatory support due to underlying central hypoventilation. However, pacing resulted in complete resolution of bradyarrhythmia‐associated desaturation episodes, with no further oxygen desaturation events temporally linked to sinus pauses.

Following confirmation of a clear temporal relationship between sinus pauses and desaturation episodes, and given the initiation of permanent pacing with stable device diagnostics, the implantable loop recorder was removed as it was no longer expected to provide additional diagnostic benefit.

**FIGURE 3:**
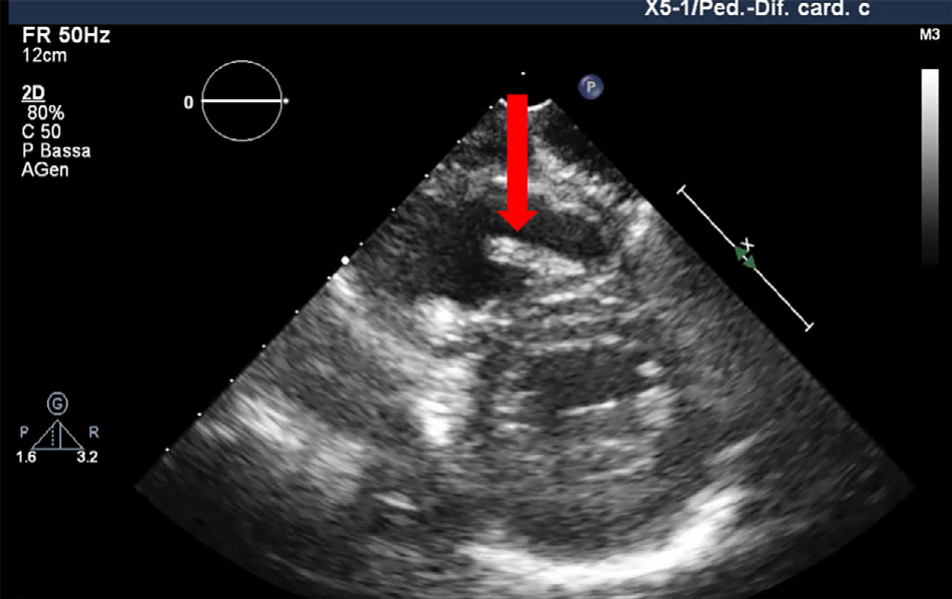
Transthoracic echocardiography showing the leadless pacemaker correctly positioned on the mid‐interventricular septum (red arrow). [Colour figure can be viewed at wileyonlinelibrary.com]

Echocardiography at discharge confirmed stable device placement (Figure 3). At one‐year follow‐up, pacing burden remained low (1%), with stable electrical parameters (sensing 9.5 mV; threshold 0.38 V at 0.24 ms; impedance 530 Ω) and an estimated remaining battery longevity of approximately 13 years. Notably, no recurrent desaturation events were recorded.

## 6 Discussion

CCHS is a rare genetic disorder of autonomic regulation characterized by impaired central respiratory drive and variable cardiovascular dysregulation. Although desaturation in CCHS is typically attributed to central apneas, our patient demonstrated the reverse sequence, prolonged sinus pauses preceding oxygen desaturation, indicating that arrhythmic events can independently trigger or worsen hypoxemia.

This observation underscores the interdependence between rhythm control and ventilatory stability in autonomic disorders. Bradyarrhythmia‐induced desaturation may perpetuate further autonomic imbalance, leading to a cycle of hypoxia and cardiac instability. In this context, pacing therapy serves a dual purpose: Preventing arrhythmic pauses and stabilizing oxygenation, thereby mitigating overall cardiorespiratory risk.

A pacemaker system was therefore indicated to prevent further desaturation and syncope risk. The successful elimination of desaturation episodes following pacing confirms the therapeutic importance of rhythm stabilization in CCHS.

## 7 Technical Considerations of Venous Access

In pediatric patients, vascular access is often the key determinant of pacing strategy [4]. In this case, small femoral veins precluded standard femoral implantation. The internal jugular route provided safe, direct access for delivery of the leadless pacemaker system and has been increasingly recognized as a feasible alternative in younger patients. While the internal jugular approach has been previously described in pediatric/adolescent patients [1, 2, 3], its use in this case was driven by patient‐specific anatomical constraints and the need for a safe, size‐appropriate venous access route.

## 8 Device Selection and Long‐Term Perspective

Given the patient's young age, device retrievability and future upgrade potential were important considerations. Newer helix‐fixated (active‐fixation) leadless pacemaker systems offer both, but at the time of intervention it was not yet commercially available in our region. The tine‐fixated (passive‐fixation) leadless pacemaker system we employed was selected due to extensive clinical experience, robust long‐term safety data, and operator familiarity, multiple factors that outweighed the theoretical advantages of retrievability in this specific case. While long‐term extraction and replacement strategies in adolescents remain somewhat unresolved, the device's proven stability and performance made it a reliable first‐line option for us in this specific case.

In addition, helix‐fixated ventricular leadless pacemakers are physically larger than tine‐fixated systems. In pediatric patients with smaller right ventricular dimensions, septal implantation of a larger device may increase the risk of interaction with the tricuspid valve apparatus, an important consideration when balancing theoretical advantages of retrievability against anatomical safety.

Given that the primary indication was sinus pauses, an atrial‐only pacing strategy using a helix‐fixated leadless atrial pacemaker (AAI) was also considered. However, this approach would have posed additional challenges in this patient. First, atrial leadless systems require stable atrial anatomy and precise fixation, which can be more demanding in pediatric patients. Second, the transjugular approach would have been technically more complex for atrial implantation, with limited clinical experience in adolescents. Finally, the potential for progression to atrioventricular conduction disease in CCHS favored a ventricular system capable of providing backup ventricular pacing if needed.

## 9 Clinical Outcome and Implications

At follow‐up, the patient remained symptom‐free, with stable pacing parameters and complete resolution of desaturation episodes. Pacing did not eliminate the need for tracheostomy‐dependent nocturnal ventilation but addressed a distinct arrhythmia‐related mechanism of hypoxemia. These results suggest that leadless pacing not only provides reliable rhythm correction but also contributes to improved oxygenation and autonomic stability in CCHS patients with combined cardiac and respiratory dysregulation.

## 10 Conclusion

This case illustrates the successful use of a leadless pacemaker implanted via the internal jugular vein in an adolescent with CCHS‐associated sinus pauses and desaturation events. Comprehensive evaluation of venous anatomy, individualized device selection, and attention to the interplay between arrhythmia and respiratory dysfunction were critical to achieving an optimal outcome.

While retrievable systems may become preferable in the future for young patients, the proven reliability and long‐term data of the device employed in this case currently make it a sound choice when procedural safety and performance are prioritized.

## Author Contributions

M.G. and G.P. contributed to patient care and data collection. H.B. and B.B. were responsible for supervision, critical revision of the manuscript, and provided expert clinical input. All authors contributed to the writing, reviewed the final manuscript, and approved its submission.

## Funding

Open Access publication was supported through the DEAL agreement between the German Rectors' Conference and Wiley.

## Conflicts of Interest

Benito Baldauf serves as a medical consultant or member of advisory boards for Abbott, Bioline Supply, Biotronik, Cablon NL, CRM Microport, Crosstec GmbH, Drugsales Limited, Kappamed, Kimal PLC, M3 Medical/Ecclipse Medical, Medival SRL, Medtronic, Philips/Spectranetics, Sintec SRL, Tauro‐Implant GmbH, Tauropharm GmbH, and Transcutan.

None of these companies had any role in the design, execution, or reporting of this case, and no specific funding was received for this work.

The other authors have nothing to declare.
